# Role of endogenous serotonin in psychedelic-like effects of psilocybin in mice

**DOI:** 10.1093/ijnp/pyaf035

**Published:** 2025-05-25

**Authors:** Ines Erkizia-Santamaría, Nerea Martínez-Álvarez, Leyre Salinas-Novoa, Jose Javier Meana, Jorge Emilio Ortega

**Affiliations:** Department of Pharmacology, University of the Basque Country UPV/EHU, Leioa, Bizkaia, Spain; Department of Pharmacology, University of the Basque Country UPV/EHU, Leioa, Bizkaia, Spain; Department of Pharmacology, University of the Basque Country UPV/EHU, Leioa, Bizkaia, Spain; Department of Pharmacology, University of the Basque Country UPV/EHU, Leioa, Bizkaia, Spain; Centro de Investigación Biomédica en Red de Salud Mental, Instituto de Salud Carlos III, Spain; Biobizkaia Health Research Institute, Barakaldo, Bizkaia, Spain; Department of Pharmacology, University of the Basque Country UPV/EHU, Leioa, Bizkaia, Spain; Centro de Investigación Biomédica en Red de Salud Mental, Instituto de Salud Carlos III, Spain; Biobizkaia Health Research Institute, Barakaldo, Bizkaia, Spain

**Keywords:** psychedelics, psilocybin, head-twitch response, serotonin, antidepressants

## Abstract

**Background:**

The psychedelic psilocybin has been posited as efficacious for the treatment of depression. However, the potential link between the intensity of acute psychedelic effects and long-term therapeutic outcomes remains undiscovered. Moreover, the impact of classical antidepressant drugs that modulate serotonergic activity on psilocybin’s effects is a clinically relevant concern. The aim of the present study was to assess serotonergic mechanisms implicated in the regulation of the intensity of the psilocybin-induced acute effects.

**Methods:**

The head-twitch response (HTR), the most translational behavioral assay to characterize the psychedelic-like effect in rodents was performed. Moreover, the role of endogenous serotonin (5-HT) on psilocybin-induced HTR was studied by *in vivo* brain microdialysis technique.

**Results:**

Maximally effective psilocybin dose (1 mg/kg) induced progressively lower HTR in heterozygous and homozygous knockout mice for serotonin 2A receptor (5HT2AR), compared to wild type. Synaptic increase of 5-HT by citalopram dose-dependently attenuated psilocybin-induced HTR after both acute and chronic dosing regimens. Conversely, depletion of 5-HT by *p-*chlorophenylalanine potentiated psilocybin-evoked HTR. Serotonin 1A receptor (5HT1AR) agonist 8-OH-DPAT dose-dependently decreased psilocybin-induced HTR, demonstrating functional interaction between 5HT2AR and 5HT1AR for psychedelic effects.

**Conclusions:**

The present findings reveal an inverse correlation between cortical 5-HT levels and the acute psychedelic-like effects of psilocybin. Consequently, the enhancement of serotonergic activity induced by prior antidepressant treatment may underlie interindividual variability in the acute response to psychedelics. Investigating these mechanisms in relation to the sustained therapeutic outcomes of psilocybin could contribute to optimizing the efficacy of psychedelic-based therapies.

## INTRODUCTION

Psychedelics powerfully alter perception, mood, and a host of cognitive processes. Despite different psychedelics having unique pharmacological profiles, extensive clinical and preclinical evidence suggests that their characteristic psychoactive effects are mediated by the activation of serotonin 2A receptors (5HT2AR).^1,2^ Subjective effects of psychedelics in humans are correlated with the occupancy of 5HT2AR in the neocortex and can be blocked by pre-administration of 5HT2AR antagonists.^2,3^ In preclinical research, the head-twitch response (HTR) is the best characterized behavioral assay to evaluate psychedelic-induced acute effects in animals. HTR has shown robust correlation with human hallucinogenic potency of psychedelic drugs,^4^ appointing it as a highly predictive paradigm, and supporting its translational relevance. In a manner analogous to the human context, HTR is blocked by pretreatment with 5HT2AR antagonists, and is absent in consequence of genetic deletion of the receptor.^1,5^ Activation of 5HT2AR located in cortical pyramidal neurons is responsible for acute psychoactive effects of psychedelics,^1^ but such effects may be modulated by drug action on other receptors.^6^ Notably, cortical regions are highly enriched in serotonin 1A receptors (5HT1AR), which abundantly co-express with 5HT2AR.^7^ According to electrophysiological and microdialysis studies, serotonin (5-HT) regulates the activity of pyramidal neurons through 5HT2AR and 5HT1AR, which respectively induce excitatory or inhibitory responses, and modulate the pyramidal output to subcortical areas in an opposite manner.^7^

In recent years, there has been a resurgence of interest in the therapeutic potential of psychedelics. Psychedelics, particularly psilocybin, have been posited as efficacious for the treatment of various neuropsychiatric illnesses, and show particularly promising results in the treatment of symptoms of depression.^8^ Clinical trials have reported significant decreases in scores of depression and anxiety scales in response to 1–3 administrations of hallucinogenic doses of psilocybin coupled with psychotherapy.^9,10^ However, these clinical results raise numerous neurobiological questions on the molecular substrate of the therapeutic mechanism of action of psilocybin. Of note, a particularly relevant matter is the potential link between the intensity of acute psychedelic effects and long-term therapeutic outcomes.^11^ Correlations have been observed between the intensity and quality of the subjective experience and therapeutic efficacy of psilocybin,^12,13^ although no direct proof exists. In fact, recent clinical evidence seems to be in disagreement.^14,15^ Therefore, mechanisms involved in the regulation of the intensity of acute psychedelic effects are highly relevant in the context of the optimization of psychedelic-based therapies.

Stemming from limitations of the currently available knowledge, concerns have been raised on potential pharmacological interactions between psilocybin and existing therapeutic agents used in the treatment of depressive disorders.^16^ Notably, selective serotonin reuptake inhibitors (SSRIs) stand out among potentially interacting drugs, and current standard of practice in psilocybin-assisted therapy trials involves discontinuation of serotonergic antidepressants several weeks before.^17^ Multiple reports have assessed the influence of classic antidepressants on the effects of psychedelics, and data suggests that antidepressant treatment may directly interfere with psychedelic-induced subjective effects, reducing the intensity of the acute psychoactive activity^14,16-19^ and may potentially impact therapeutic outcomes. However, the absence of mechanistic studies hinders the understanding of the pharmacological basis of this phenomenon.

Against this background, the aim of the present study was to evaluate the role of potential modulatory mechanisms of 5HT2AR activation-induced HTR in mice. The maximal dose of psilocybin for HTR in mouse (1 mg/kg) was previously characterized in a dose–response study,^5^ and selected for the present work. Research suggests that the quality and intensity of the acute psychedelic experience may be critical for triggering the long-term therapeutic effects of psilocybin. Therefore, this study focuses on the modulatory mechanisms of commonly prescribed antidepressants on psilocybin-induced psychedelic effects. With these purposes, diverse strategies were employed. First, we characterized the role of 5HT2AR on psilocybin-induced HTR in wild type (WT), heterozygous (Het, htr2a^+/−^), and knockout (KO, htr2a^−/−^) mice for 5HT2AR. For the assessment of the role of endogenous 5HT2AR ligand 5-HT on psilocybin-induced HTR, neurotransmitter brain concentrations were pharmacologically increased or decreased by means of SSRI citalopram or irreversible tryptophan hydroxylase (TPH) inhibitor *p*-chlorophenylalanine (PCPA) administration, respectively. Finally, in order to evaluate 5HT1AR-mediated mechanism, different doses of 5HT1AR agonist 8-OH-DPAT were administered prior to psilocybin.

## EXPERIMENTAL PROCEDURES

### Animals

Adult male WT C57BL/6J mice (8 weeks old) were purchased from Envigo. Heterozygous (Het, htr2a^+/−^) mice (C57BL6 background) were purchased from Model Organisms and bred in-house to obtain male and female 5HT2AR knockout (KO) (htr2a^−/−^). Mice were housed under standard laboratory conditions in compliance with ARRIVE guidelines,^20^ on a 12-hour light/dark cycle, at room temperature (22°C–24°C), with food and water *ad libitum*. The total number of animals used for this study was 119, and group sizes per experiment were 4–9. Animal care and experimental protocols were carried out in accordance with principles of animal care established by the EU Directive 2010/63/EU and in agreement with Spanish legislation (Royal Decree 53/2013), and were approved by the UPV/EHU Ethical Board of Animal Welfare (CEEA: M20_2020_014).

### Drugs

Psilocybin [3-[2-(dimethylamino)-ethyl]-1H-indol-4-ol dihydrogen phosphate] was obtained from THC Pharm and dissolved to 0.2 mg/mL in saline solution (SS, 0.9% NaCl). Citalopram [1-[3-(dimethylamino)propyl]-1-(4-fluorophenyl)-3H-2-benzofuran-5-carbonitrile] hydrobromide was obtained from Tocris and dissolved to 4 and 8 mg/mL in SS. *p-*chlorophenylalanine (PCPA) [2-amino-3-(4-chlorophenyl)propanoic acid] was obtained from Sigma-Aldrich and dissolved in vehicle (0.9% NaCl, 0.4% Tween 20) to 40 mg/mL. MDL11939 [α-phenyl-1-(2-phenylethyl)-4-piperidinemethanol] was obtained from Sigma-Aldrich and dissolved to 0.1 mg/mL in SS with a minimal amount of glacial acetic acid to facilitate dissolution. 8-OH-DPAT [7-(dipropylamino)-5,6,7,8-tetrahydronaphthalen-1-ol] hydrobromide was obtained from Sigma-Aldrich and dissolved in SS to 0.02 and 0.2 mg/mL. WAY100635 [*N*-[2-[4-(2-methoxyphenyl)- 1-piperazinyl]ethyl]-*N*-2-pyridinylcyclohexanecarboxamide maleate] was obtained from Tocris and dissolved to 0.1 mg/mL in SS.

### Pharmacological Treatments

Pretreatments with citalopram (20 and 40 mg/kg), WAY100635 (1 mg/kg), 8-OH-DPAT (0.1 and 1 mg/kg), MDL11939 (1 mg/kg), or vehicle were administered 30 minutes prior to most effective HTR-inducing dose of psilocybin (1 mg/kg) or saline.^5^ For 5-HT depletion, PCPA administration protocol was adapted from previous works.^21-23^ PCPA (400 mg/kg) was administered 24 hours before psilocybin (1 mg/kg) or saline. For chronic treatment with citalopram (40 mg/kg), the drug was administered for 14 days prior to HTR or microdialysis experiments. All drugs were administered by intraperitoneal (i.p.) injection (5–10 mL/kg).

### Head-Twitch Response Assessment

HTR was assessed in an open field chamber (43 × 43 × 43 cm), and recorded with a camera device (ASUS Zenfone 3), as previously described.^5^ HTR was manually quantified by a trained and blinded observer between minutes 5 and 25 after psilocybin administration. Light intensity was established and fixed at 60 lux. The open fields were thoroughly cleaned with 70% ethanol and were left to fully dry between test sessions.

### Stereotaxic Surgery and Microdialysis Procedures

Mice were anesthetized with isoflurane gas using a CA-ECA20 Anesthesia Trolley System equipment, and placed in a stereotaxic frame (David Kopf Instruments) with the head horizontally immobilized. An incision into the scalp was made to reveal lambda and bregma suture points. One microdialysis probe (2.0 × 0.25 mm) was implanted in the prefrontal cortex (PFC) (anterior–posterior (A/P): +2.0 mm; medial–lateral (M/L): +0.3 mm; dorsoventral (D/V): −3.3 mm, coordinates taken from bregma). These coordinates were chosen using the Paxinos and Franklin atlas.^24^ Probes were fixed to the skull with dental cement and protected with a plastic cover to prevent damage. Once recovered from surgery, mice were housed individually with water and food *ad libitum*.

All the microdialysis experiments were performed between 20 and 24 hours after probe implantation, as previously described.^25^ Sampling was carried out using a Raturn Microdialysis System (BASi), with animals freely moving in the cage. After recovery, probes were connected to the system and artificial cerebrospinal fluid solution (148 mM NaCl, 2.7 mM KCl, 1.2 mM CaCl_2_, and 0.85 mM MgCl_2_ with pH of 7.4, adjusted with 1 mM K_2_HPO_4_) was perfused through the probes at a rate of 1 μL/min incessantly during the whole experiment. Following 1 hour of stabilization, dialysate samples were collected every 35 minutes in refrigerated vials. Six samples were obtained before drug administration.

### Chromatographic Analysis and Determination of 5-HT Concentration in Dialysate

5-HT concentration in dialysate samples was immediately determined using HPLC equipment with amperometric detection (VT-03 cell, Decade II, Antec Scientific) at an oxidizing potential of 0.30 V.^26^ Separation was carried out with a mobile phase composed of 50 mM phosphoric acid, 0.1 mM EDTA, 8 mM NaCl, 500 mg/L sodium octyl sulfate, and 19% methanol. pH was adjusted to 6.0 with NaOH 6 N. The mobile phase was filtered and delivered at a flow rate of 0.2 mL/min in an ALF-215, 2.1 × 150 mm, C18 (Antec Scientific) chromatographic column by a model 1100 pump (Agilent Technologies) after degasification (Agilent Technologies model 1100 degasser). 35 µL volume of each sample was placed in the refrigerated autosampler and a volume of 30 µL was injected in the HPLC system. The peak area of 5-HT was integrated by a Chemstation plus Software (Hewlett Packard Ltd.). The detection limit of the assay was approximately 0.05 nM.

### Tissue Processing and Chromatographic Analysis for the Quantification of Monoamine Levels

Immediately after the HTR assessment, the right brain cortex (R Ctx) and left brain cortex (L Ctx) were harvested and quickly frozen until processing. Samples were defrosted, then homogenized in a cold buffer (0.1 N perchloric acid with 100 µM EDTA, 15 µL/mg of tissue) using a T-10 basic Ultra-Turrax (IKA). Homogenates were centrifuged at 20 817 RCF/*g*-force for 15 minutes at 4 °C. Supernatants were filtered (Costar Spin-X Centrifuge filters 0.22 μm, Sigma-Aldrich) at 1000 RCF/*g*-force for 5 minutes at 4 °C. From the final filtrates, 40 µL of each sample was separated for immediate analysis. Cortical monoamine content was evaluated by HPLC. Separation was carried out with a mobile phase composed of 50 mM phosphoric acid, 0.1 mM EDTA, 8 mM NaCl, 500 mg/L sodium octyl sulfate, and 12% methanol. pH was adjusted to 6.0 with NaOH 6 N, as previously described.^25^ The detection limit of the assay was approximately 0.1 nM.

### Statistical Analysis

For HTR evaluation, data were analyzed using 1-way analysis of variance (ANOVA). For microdialysis experiments 2-way repeated-measures ANOVA was used, with treatment groups as the independent variable (*F*_drug_), and time as the repeated measure factor (*F*_time_). Significant interaction between variables (*F*_*i*_) was followed by Bonferroni post hoc test. Basal values between groups and pre- and post-administration values were compared by unpaired Student’s *t*-test or 1-way ANOVA, followed by Bonferroni post hoc test. For monoamine content evaluation, data were analyzed using unpaired *t-t*est. Simple linear regression was used to analyze correlation between tissue monoamine concentration and HTR. All results are shown as mean ± SEM. Reduction/increase of HTR and monoamine concentrations were calculated as the percentage of the mean of the group of reference. In all cases, statistical significance was considered when *P* < .05. Data were analyzed using GraphPad Prism 10.1.

## RESULTS

### Psilocybin-Induced HTR in WT, Het, and KO Mice for 5HT2AR

**Figure 1.**
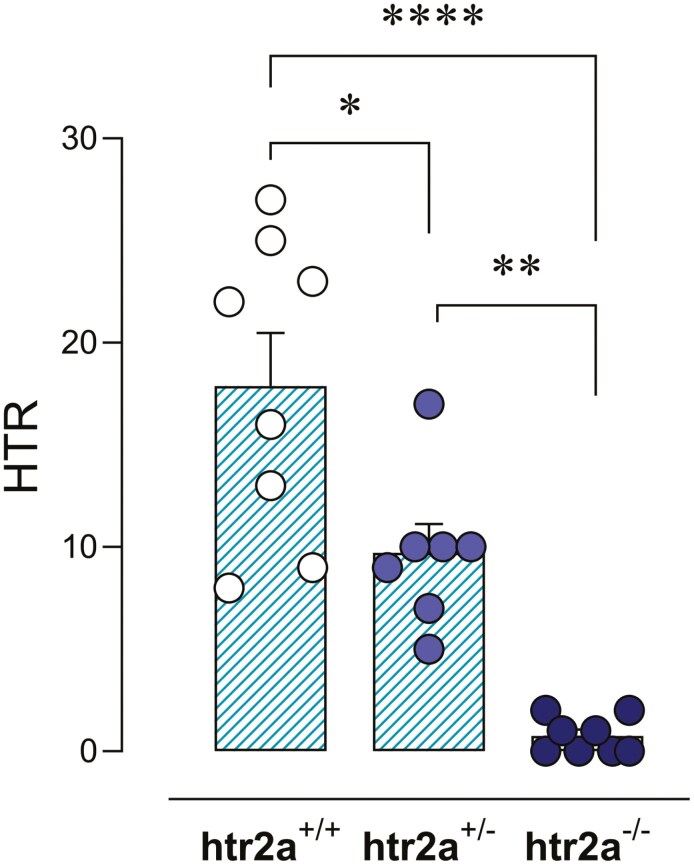
Psilocybin (1 mg/kg, intraperitoneal [i.p.])-induced head-twitch response (HTR) on wild type (WT) (htr2a^+/+^), heterozygous (htr2a^+/−^), and knockout (KO) (htr2a^−/−^) mice for 5HT2AR. One-way analysis of variance (ANOVA) followed by Bonferroni post hoc test. *P < .05; **P < .01; ****P < .0001.

Psilocybin induced significantly different HTR in WT, Het, and KO mice. One-way ANOVA analysis confirmed a significant effect of genotype (*F*(2,20) = 24.99; *P* < .0001), and Bonferroni post hoc test revealed significantly lower HTR in Het (−46% ± 8%; *t* = 3.25; *P* < .05), and KO (−96% ± 2%; *t* = 7.07; *P* < .0001) compared to WT mice (Figure 1, Table S1). Psilocybin-induced HTR was not affected by sex (Figure S1).

### Role of Endogenous 5-HT in Psilocybin-Induced HTR

**Figure 2.**
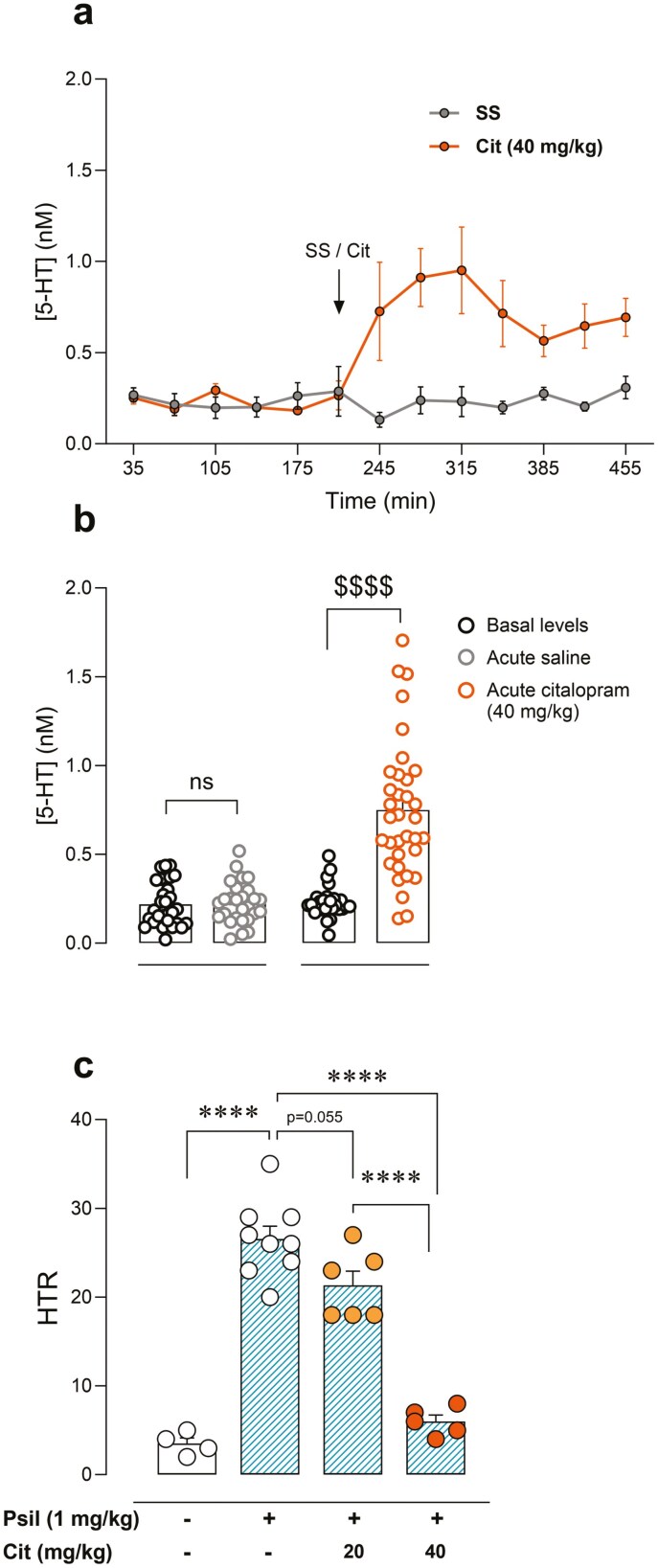
(A) Effect of systemic citalopram (40 mg/kg, intraperitoneal [i.p.]) or saline solution (SS) administration in extracellular 5-HT concentration in prefrontal cortex (PFC) of mice. (B) Extracellular concentrations of 5-HT in PFC of mice under basal conditions or following drug administration (SS or citalopram). Unpaired *t*-test, ^$$$$^*P* < .0001. ns, nonsignificant. (C) Effect of citalopram (20 or 40 mg/kg, i.p.) on psilocybin (1 mg/kg, i.p.)-induced head-twitch response (HTR). One-way analysis of variance (ANOVA) followed by Bonferroni post hoc test. ****P < .0001.

To decipher the influence of 5-HT on psilocybin-induced HTR, extracellular concentrations of the endogenous ligand were augmented by citalopram administration 30 minutes before psilocybin. First, we confirmed that the systemic administration of citalopram induced a significant increase in extracellular 5-HT concentrations in the PFC (*F*_drug_(1, 9) = 8.79, *P* < .05; *F*_time_(2.23, 15.83) = 5.11, *P* < .05; *F*_*i*_(12, 85) = 6.01, *P* < .0001) (Figure 2A), (pre- vs post-administration, *t* = 6.97, *P* < .0001) (Figure 2B). Then we studied the effect of citalopram pretreatment on psilocybin-induced HTR. Citalopram exerted a dose-dependent decrease in psilocybin-induced HTR (*F*(3, 20) = 63.92, *P* < .0001) (Figure 2C). Post hoc comparison between vehicle + psilocybin and citalopram (20 mg/kg) + psilocybin groups revealed nonsignificant HTR reduction (−20% ± 6%; *t* = 2.88, *P* = .055). Furthermore, comparison between vehicle + psilocybin vs citalopram (40 mg/kg) + psilocybin groups indicated a greater reduction in HTR in consequence of citalopram pretreatment (−77% ± 3%; *t* = 10.72, *P* < .0001). Citalopram, when administered alone, demonstrated no effect on HTR (Figure S2).

**Figure 3.**
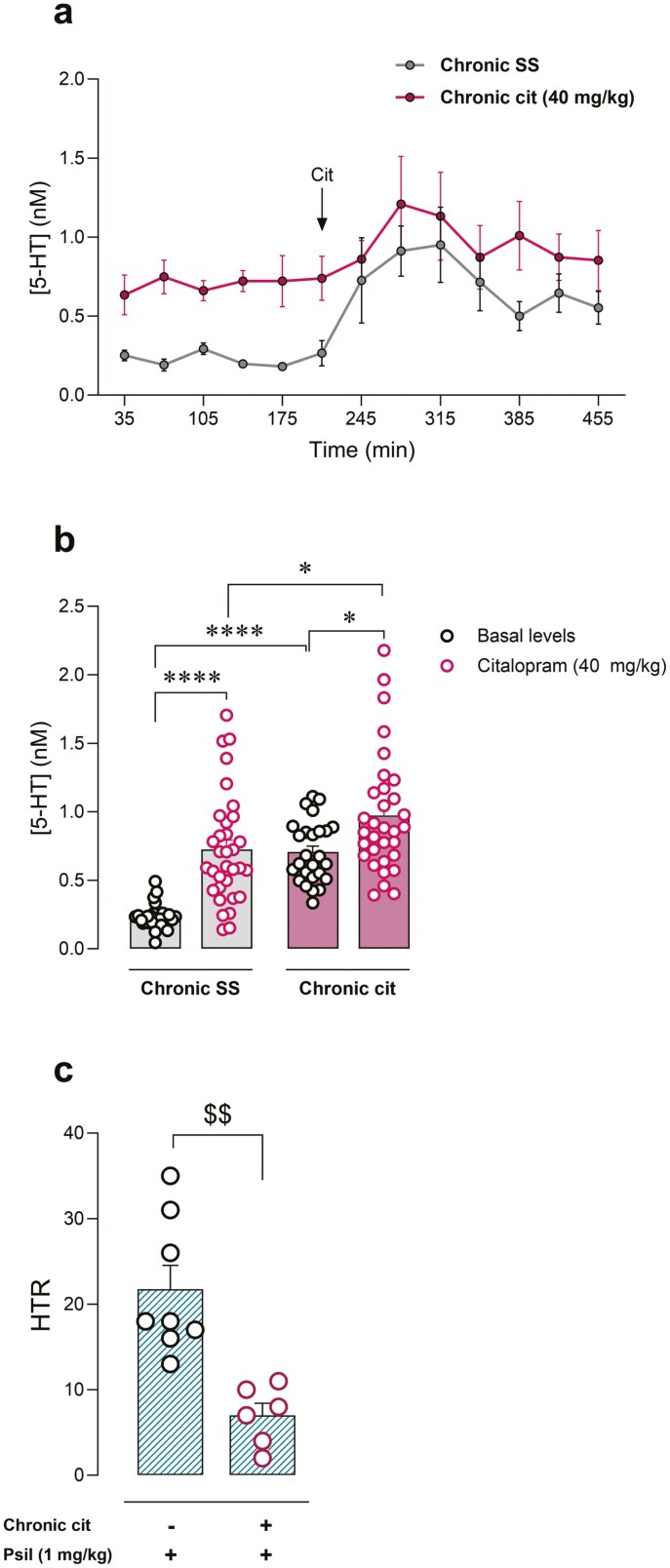
(A) Effect of chronic dosing regimen of systemic citalopram (40 mg/kg, intraperitoneal [i.p.], 14 days) administration in basal extracellular 5-HT concentration in prefrontal cortex (PFC) of mice, and effect of acute citalopram (40 mg/kg, i.p.) administration. (B) Extracellular concentrations of 5-HT in PFC of mice chronically treated with saline solution (SS) or citalopram (40 mg/kg, i.p., 14 days), under basal conditions or following acute citalopram (40 mg/kg, i.p.) administration. One-way analysis of variance (ANOVA) followed by Bonferroni post hoc test. *P < .05; ****P < .0001. (C) Effect of chronic dosing regimen with citalopram (40 mg/kg, i.p., 14 days) or SS on psilocybin (1 mg/kg, i.p.)-induced head-twitch response (HTR). Unpaired *t*-test, ^$$^*P* < .01.

Then we evaluated if such mechanisms operate in a similar manner after a chronic dosing regimen. We first confirmed that chronic treatment with citalopram (40 mg/kg, i.p., 14 days) induced a significant increase in basal extracellular 5-HT concentrations in PFC, compared to chronic saline (*t* = 5.30, *P* < .0001) (Figure 3A,B). Moreover, acute citalopram administration also induced a significant increase in extracellular 5-HT in PFC in chronically treated animals (pre- vs post-administration, *t* = 3.04, *P* < .05) (Figure 3A,B). Next, we assessed the effect of chronic citalopram treatment on psilocybin-induced HTR. In order to avoid acute effect, 24 hours after the last citalopram dose psilocybin was administered. Chronic citalopram-treated mice showed a significantly lower psilocybin-evoked HTR when compared to chronic saline animals (−68% ± 7%, *t* = 4.23, *P* < .01) (Figure 3C).

**Figure 4.**
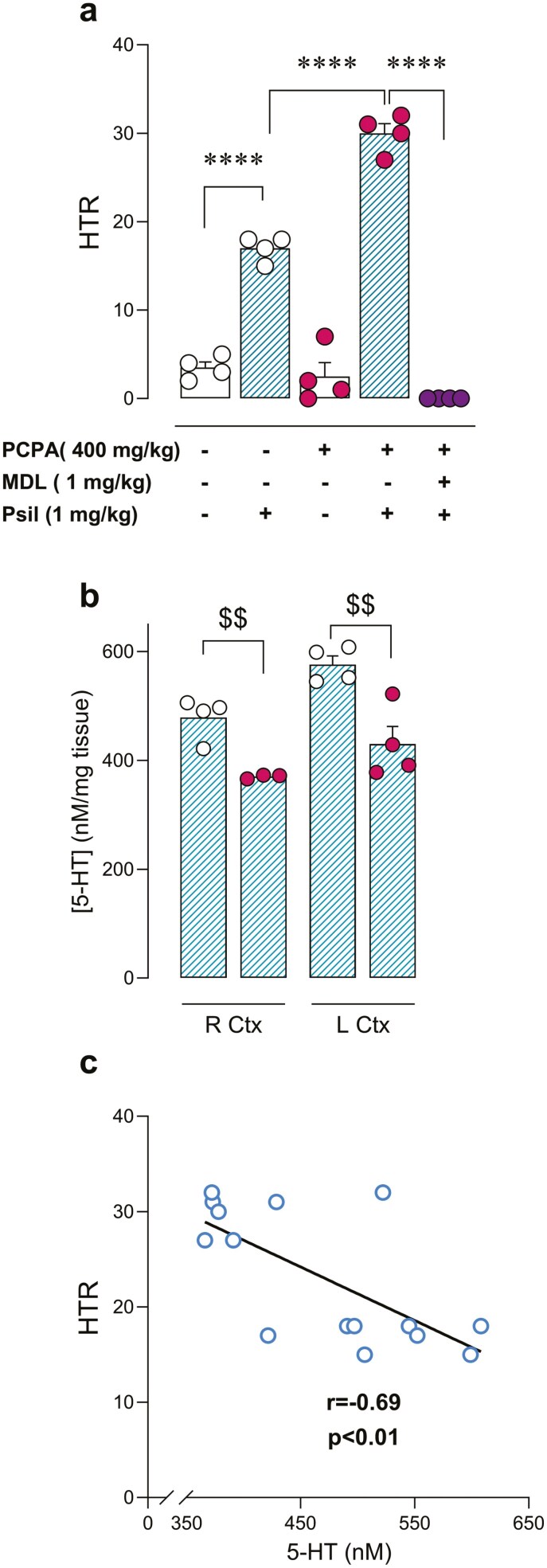
(A) Effect of *p*-chlorophenylalanine (PCPA) (400 mg/kg, intraperitoneal [i.p.]) on psilocybin (1 mg/kg, i.p.)-induced head-twitch response (HTR), and blockade by pre-administration with 5HT2AR antagonist MDL11939 (1 mg/kg, i.p.). One-way analysis of variance (ANOVA) followed by Bonferroni post hoc test. ****P < .0001. (B) 5-HT tissue levels in right brain cortex (R Ctx) and left brain cortex (L Ctx) of vehicle/PCPA (400 mg/kg, i.p.) pretreated mice. Unpaired *t-*test. ^$$^*P* < .01. (C) Correlation between cortical 5-HT and psilocybin-induced HTR.

Next, 5-HT depletion was also induced by pretreatment with the TPH inhibitor PCPA. Twenty-four hours post-PCPA (400 mg/kg, i.p.) or vehicle administration, psilocybin (1 mg/kg, i.p.), or saline was administered and HTR was evaluated. One-way ANOVA revealed a significant difference between the compared groups (*F*(4, 15) = 179.40, *P *< .0001). Post hoc test showed significant increase in HTR in vehicle + psilocybin versus PCPA + psilocybin groups (+76% ± 6%; *t* = 9.69, *P* < .0001) (Figure 4A). Pre-administration of 5HT2AR antagonist MDL11939 (1 mg/kg, i.p.) 30 minutes before psilocybin in PCPA-treated animals completely blocked psilocybin-induced HTR (*t* = 22.36, *P* < .0001) (Figure 4A). These data provide evidence that psilocybin-triggered HTR potentiation under PCPA treatment is a consequence of 5HT2AR activation. In these animals, 5-HT concentrations were also determined to verify PCPA-induced 5-HT depletion (24-hour post-PCPA administration). PCPA selectively reduced tissue 5-HT content in comparison to a vehicle in R Ctx (−23% ± 0.5%; *t* = 4.72, *P* < .01) and L Ctx (−25% ± 6%; *t* = 4.03, *P* < .01) (Figure 4B). Moreover, a significant inverse correlation was found between cortical 5-HT concentration and HTR elicited (*r* = −0.692, *P* < .01) (Figure 4C). In contrast, PCPA pretreatment did not modify cortical DA or NA, and the concentration of neither correlated with HTR elicited (Figure S3).

### Role of 5HT1AR Agonism in Psilocybin-Induced HTR

**Figure 5.**
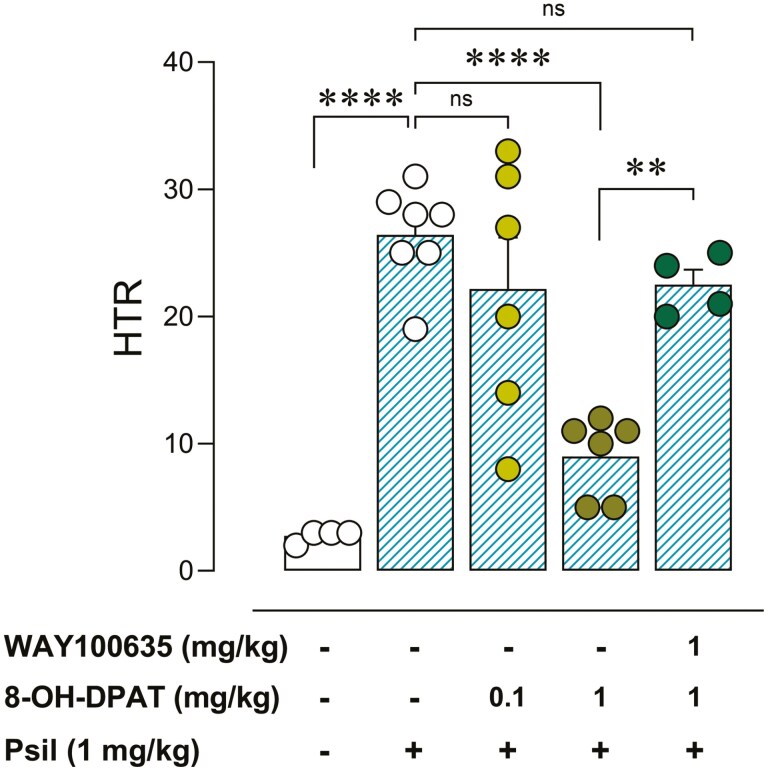
Effect of 8-OH-DPAT (0.1 or 1 mg/kg, intraperitoneal [i.p.]) on psilocybin (1 mg/kg, i.p.)-induced head-twitch response (HTR), and reversal of partial blockade exerted by 8-OH-DPAT (1 mg/kg) by co-administration with 5HT1AR antagonist WAY100635 (1 mg/kg, i.p.). One-way analysis of variance (ANOVA) followed by Bonferroni post hoc test. **P < .01; ****P < .0001. ns, nonsignificant.

The influence of 5HT1AR activation was investigated through pretreatment with 5HT1AR agonist 8-OH-DPAT 30 minutes prior to psilocybin. 8-OH-DPAT pretreatment attenuated psilocybin-induced HTR (*F*(4, 22) = 17.70; *P* < .0001). Bonferroni post hoc test showed no significant difference in HTR between vehicle + psilocybin versus 8-OH-DPAT (0.1 mg/kg) + psilocybin (−17% ± 15%; *t* = 1.41, *P* > .05). However, a significant reduction in HTR was revealed between vehicle + psilocybin versus OH-DPAT (1 mg/kg) + psilocybin groups (−66% ± 5%; *t* = 5.76, *P* < .0001). Such reduction was rescued by pretreatment with 5HT1AR antagonist WAY100635 (1 mg/kg) (*t* = 3.85, *P* < .01) (Figure 5). Additionally, we have ruled out a role for 5HT2CR in the psilocybin-mediated effect at the dose of 1 mg/kg i.p. (see Figure S4 for details).

## DISCUSSION

In the present study, we have explored different mechanisms intervening in the modulation of psychedelic-like effect induced by psilocybin. We first confirmed the well-known critical implication of 5HT2AR in psilocybin-evoked HTR, as KO (htr2a^−/−^) mice exhibited no significant response, in accordance with previous results.^6^ Interestingly, Het (htr2a^+/−^) mice showed significantly lower HTR compared to WT littermates, conceivably due to the lower degree of receptor expression, but significantly higher response than homozygous KO mice. This result does not align with one previously reported experiment in which the loss of a single allele in htr2a^+/−^ was not seen to interfere with psychedelic-induced HTR.^1^ Recently, it has been reported that the psychedelic DOI preferentially activates a subset of neuronal subtypes in the mPFC^27^ and that pyramidal tract neurons of the frontal cortex, which express 5HT2AR, play essential roles in psilocybin’s actions.^28^ Thus, further investigation in cell-specific 5HT2AR KO animal models is warranted.

In the present work, we hypothesized that the endogenous ligand 5-HT could directly interfere with psilocybin on its target receptor for psychedelic effects, by competitive antagonism. In this regard, the intensity of the psilocybin-induced psychedelic effect may serve as a potential biomarker phenotype for long-term therapeutic response. Likewise, the degree of serotonergic activity prior to psychedelic treatment may account for the interindividual variability in long-term clinical response. In this sense, in the present study, we have observed that the increase of extracellular 5-HT concentrations after citalopram administration was accompanied by an inhibitory effect on psilocybin-induced psychedelic effect. Moreover, the present study demonstrates that these mechanisms function similarly whether an acute increase in 5-HT is induced immediately before psilocybin administration or when extracellular 5-HT concentrations are increased through a chronic dosing regimen. Interestingly, it has been reported that serotonin transporter KO mice exhibit increased extracellular 5-HT concentrations,^29^ which may be relevant to the lack of psilocybin-induced HTR observed at the same dose tested in the present study in these KO animals.^30^ Furthermore, the opposite effect was induced by the depletion of 5-HT content. Pre-administration of the TPH inhibitor PCPA was able to enhance psilocybin-induced HTR, in accordance with the posited competition-based mechanism. Interestingly, the psychedelic effect was completely blocked by the administration of the 5HT2AR antagonist MDL11939, indicating that the increase of HTR in PCPA-treated animals is also induced by 5HT2AR-dependent mechanisms. It is relevant to note that HTR elicited by psilocybin negatively correlated with 5-HT content in the brain cortex, the area that mediates 5HT2AR-dependent psychedelic effects.^1^ Altogether, the present results identify in detail that the mechanism of this pharmacodynamic interaction between citalopram and psilocybin occurs indirectly through a previous increase of synaptic 5-HT in the brain cortex.

In light of the observed effect, it is crucial to consider the potential for a drug–drug pharmacological interaction. However, these alternative mechanisms are very improbable in view of the existence of neurobiological mechanisms operating acutely. Notably, citalopram lacks affinity for 5HT2AR^31^ and the administration of citalopram or PCPA as monotherapy did not induce HTR, suggesting that a direct pharmacodynamic interaction with psilocybin is unlikely. In addition, citalopram is primarily metabolized in the liver via the cytochrome P450 (CYP450) enzymatic system^32^ whereas a significant proportion of psilocybin metabolism is performed by glucuronidation through UDP-glucuronosyltransferases or by oxidation within neuronal tissue via monoamine oxidase.^11^ Furthermore, the administration protocol for PCPA is performed 24 hours prior to psilocybin administration, which further reinforces the rationale for ruling out a pharmacokinetic interaction.

An alternative mechanism to elucidate changes in HTR as a consequence of modulation of the serotonergic tone could be an indirect functional antagonism of psychedelic effects by causing an imbalance of 5HT1AR/2AR activation, as previously proposed elsewhere.^33^ In line with preclinical data, clinical studies have also observed notable interactions between SSRI antidepressants and psychedelics. Clinical trials and observational studies have reported that SSRIs may reduce the intensity of psychedelic experience and weaken some of the acute effects of psilocybin,^16-18^ and this observation also extends to other psychedelics (ie, LSD, mescaline, etc.).^14,19^ Therefore, preclinical and clinical evidence suggests that acute psychedelic effects can be significantly impacted by previous potentiation of serotonergic neurotransmission. Scarce long-term data so far seem to indicate that concomitant use of SSRI antidepressants and psychedelics does not hinder the therapeutic potential of the latter.^14,15^ This approach, however, is not aligned with the theory that the intensity of psychedelic experience correlates with long-term benefits.^12,13^ Albeit the question of whether co-administration of SSRIs and psychedelics could reduce the efficacy of psychedelic-based therapies remains open, and a larger number of long-term studies of clinical outcomes and preclinical behavioral data are required to provide definitive clarity on the matter.

In the search for the pharmacological mechanism responsible for the suppression of 5HT2AR activation-induced HTR, 5HT1AR is posited as a strong candidate. In the present work, pretreatment with selective 5HT1AR agonist 8-OH-DPAT dose-dependently attenuated psilocybin-induced HTR. Previous reports have described that pre-administration of 8-OH-DPAT decreases HTR induced by several psychedelics in mice.^34,35^ On the contrary, selective 5HT1AR antagonist WAY100635 can potentiate HTR at doses of psychedelic above maximal for HTR,^34^ but not at maximally active dose of psilocybin.^5^ Moreover, another report found that the descending limb of HTR dose–response curves of several psychedelics was concurrent with the appearance of 5HT1AR-mediated pharmacological effects (ie, hypothermia),^36^ suggesting that 5HT1AR activation counteracts HTR. Regarding human studies, pretreatment of participants with 5HT1AR partial agonist buspirone has been reported to reduce psychedelic effects of psilocybin,^33^ while 5HT1AR antagonist pindolol increased hallucinogenic effects of N,N-dimethyltryptamine (DMT).^37^ Current and previous evidence therefore suggests that activation of 5HT1AR exerts functional antagonism on 5HT2AR-mediated effects.^38^ Given the alleged role of 5HT1AR in long-term changes induced by psychedelics (ie, neuroplasticity),^39^ future clinical and preclinical works should examine the specific role of such molecular target in modulating acute and long-term therapeutic effects of psychedelics at clinically relevant doses.

Considering the complex, multitargeted pharmacodynamic profile of psilocin, several possibilities arise as potential candidates for the therapeutic mechanism of action. However, an ongoing debate on the role of the intensity and quality of psychedelic experience and the link to long-term therapeutic outcomes remains. The present work helps further extend the knowledge on the pharmacological mechanisms underlying the acute psychoactive effects of psilocybin and provides mechanistic insight of high translational value. Data here presented suggest that a functional interaction between 5HT2AR and 5HT1AR counterbalances psychedelic activity. Such mechanisms could directly or indirectly (via endogenous 5-HT) modulate the intensity of acute psychedelic effects. Follow-up studies will be critical to elucidate whether enhancing or dampening the psychedelic experience improves the long-term efficacy of psychedelic-based therapies. In fact, recent evidence has suggested that the HTR and anxiolytic effects of psychedelics can be dissociated.^27^ This matter is pivotal for promoting clinical scalability by maximizing the potential benefits of psychedelic treatments, while reducing unwanted effects and treatment costs. Likewise, the activity of afferent serotonergic pathways to PFC and interactions between 5HT2AR- and 5HT1AR-dependent processes could be involved in the pathophysiological mechanisms of hallucinations in psychiatric diseases. Thus, additional studies investigating the neurobiological mechanisms underlying these phenomena are needed.

### Limitations of the Study

The present study focused on evaluating the interaction between citalopram and psilocybin at the maximum HTR-inducing dose,^5^ addressing exclusively acute neurochemical and behavioral mechanisms. Therefore, this work enables the identification of a pharmacodynamic interaction mechanism between 5-HT and the psychedelic-like effect of psilocybin. Further studies are required to confirm whether such mechanisms are present when combining different doses of psilocybin or other psychedelics with various serotonergic-enhancing drugs. The assessment of such interactions regarding their long-term neurochemical and behavioral effects is a question that must be addressed in future research.

As previously outlined, there is significant controversy regarding whether the induction of psychedelic experiences is necessary to achieve antidepressant therapeutic outcomes.^40,41^ It is conceivable that certain 5HT2AR agonist drugs lacking psychedelic activity may still induce long-term neuroplastic and therapeutic effects, as suggested by some authors.^42^ In this sense, hallucinogenic and non-hallucinogenic 5HT2AR agonists, although binding to the same orthosteric site, differ in their functional outcomes, which extend beyond the canonical G_q/11_-mediated signaling pathway and include differential interactions with other G protein subtypes and β-arrestin isoforms.^43,44^ These observations suggest that the therapeutic efficacy of 5HT2AR activation may not be solely dependent on the subjective psychedelic experience, but rather on the specific intracellular signaling pathways engaged. In this regard, regardless of this debate, the presence of enhanced cortical serotonergic activity—whether intrinsic to the individual or induced through specific pharmacological treatments—could attenuate the activation of transduction pathways to which the 5HT2AR is coupled, potentially influencing any long-term mechanisms required for therapeutic action. The complexity increases when considering that the activation of other serotonergic receptors, resulting from elevated extracellular 5-HT concentration, may modulate transduction pathways in a manner opposite to those activated by potential 5HT2AR agonist drugs (both psychedelic and non-psychedelic). These complexities highlight significant gaps in our current understanding and underscore the need for continued investigation. Future studies should aim to clarify these mechanisms and their implications for therapeutic strategies.

## Supplementary Material

pyaf035_suppl_Supplementary_Table_S1_Figures_S1-S4

## Data Availability

The data sets generated for this study are available on request to the corresponding author.
