# Supplementary material for: Role of endogenous serotonin in psychedelic-like effects of psilocybin in mice
Source: Int J Neuropsychopharmacol. 2025 May 25;28(6):pyaf035. doi: 10.1093/ijnp/pyaf035 (PMC12202319; doi:10.1093/ijnp/pyaf035)
Supplement: pyaf035_suppl_Supplementary_Table_S1_Figures_S1-S4 [file pyaf035_suppl_supplementary_table_s1_figures_s1-s4.docx]

**Table S1**. HTR elicited by psilocybin (1 mg/kg) in different genotypes for 5HT2AR, and by different pre-treatments and treatments in wild-type mice. *Statistically significant difference relative to group of reference (100%).

| **Genotype** | **Treatment (dose)** | **HTR mean ± SEM** | **HTR (% wild-type)** |
| --- | --- | --- | --- |
| Wild-type (htr2a^+/+^) | Psilocybin (1 mg/kg) | 17.88 ± 2.61 | 100% |
| Heterozygous (htr2a^+/-^) | Psilocybin (1 mg/kg) | 9.71 ± 1.41 | 54 ± 8%* |
| Knockout (htr2a^-/-^) | Psilocybin (1 mg/kg) | 0.75 ± 0.31 | 4 ± 2%* |
| **Pre-treatment (dose)** | **Treatment (dose)** | **HTR (mean ± SEM)** | **HTR (% relative to psilocybin 1 mg/kg)** |
| Vehicle | Saline solution (5 mL/Kg) | 3.50 ± 0.65 | - |
| Vehicle | Psilocybin (1 mg/kg) | 26.56 ± 1.43 | 100% |
| Citalopram (20 mg/kg) | Psilocybin (1 mg/kg) | 21.33 ± 1.59 | 80 ± 6% |
| Citalopram (40 mg/kg) | Psilocybin (1 mg/kg) | 6.00 ± 0.71 | 23 ± 3%* |
| Chronic vehicle | Psilocybin (1 mg/kg) | 21.75 ± 2.80 | 100% |
| Citalopram (40 mg/kg, chronic dosing regimen) | Psilocybin (1 mg/kg) | 7.00 ± 1.41 | 32 ± 7%* |
| Vehicle | Saline solution (5 mL/Kg) | 3.50 ± 0.65 | - |
| Vehicle | Psilocybin (1 mg/kg) | 17.00 ± 0.71 | 100% |
| PCPA (400 mg/kg) | Saline solution (5 mL/Kg) | 2.50 ± 1.56 | - |
| PCPA (400 mg/kg) | Psilocybin (1 mg/kg) | 30.00 ± 1.08 | 176 ± 6%* |
| PCPA (400 mg/kg) +  MDL11939 (1 mg/kg) | Psilocybin (1 mg/kg) | 0.00 ± 0.00 | 0 ± 0 %* |
| Vehicle | Saline solution (5 mL/Kg) | 2.75 ± 0.25 | - |
| Vehicle | Psilocybin (1 mg/kg) | 26.43 ± 1.48 | 100% |
| 8-OH-DPAT (0.1 mg/kg) | Psilocybin (1 mg/kg) | 22.17 ± 4.05 | 83 ± 15% |
| 8-OH-DPAT (1 mg/kg) | Psilocybin (1 mg/kg) | 9.00 ± 1.30 | 34 ± 5%* |
| WAY100635 (1 mg/kg) +  8-OH-DPAT (1 mg/kg) | Psilocybin (1 mg/kg) | 22.50 ± 1.19 | 85 ± 5% |


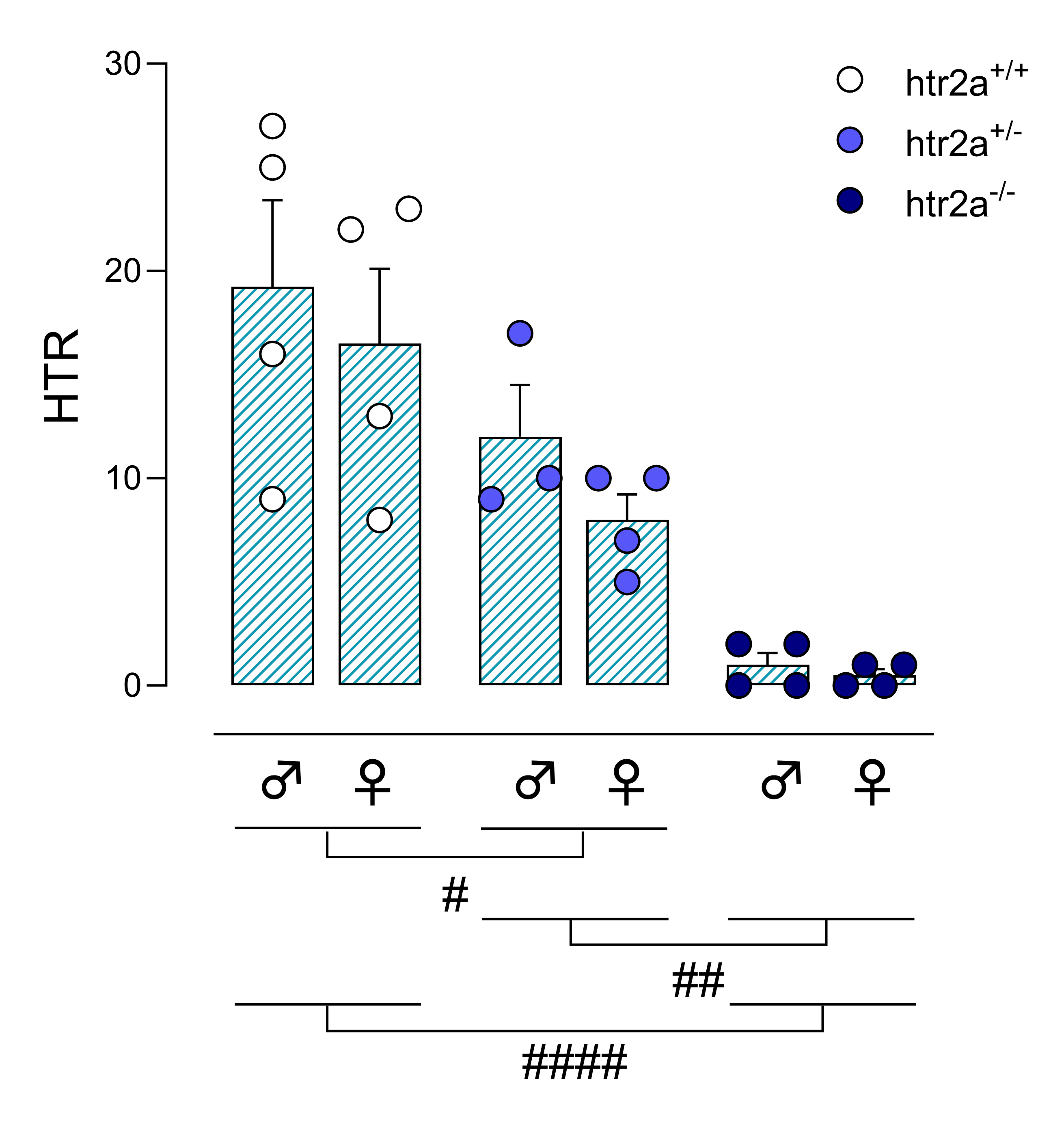


**Figure S1.** Psilocybin (1 mg/kg, i.p.)-induced HTR on WT (htr2a^+/+^), heterozygous (htr2a^+/-^), and KO (htr2a^-/-^) male (♂) and female (**♀**) mice for 5HT2AR. Two-way ANOVA revealed significant effect of genotype (F_gen_(2,17)=23.41, p<0.0001). However, psilocybin-induced HTR was not affected by sex (F_sex_(1,17)=1.32, p>0.05), and no significant interaction between sex and genotype was found (F_i_ (2,17)=0.23, p>0.05).

**Figure S2.**
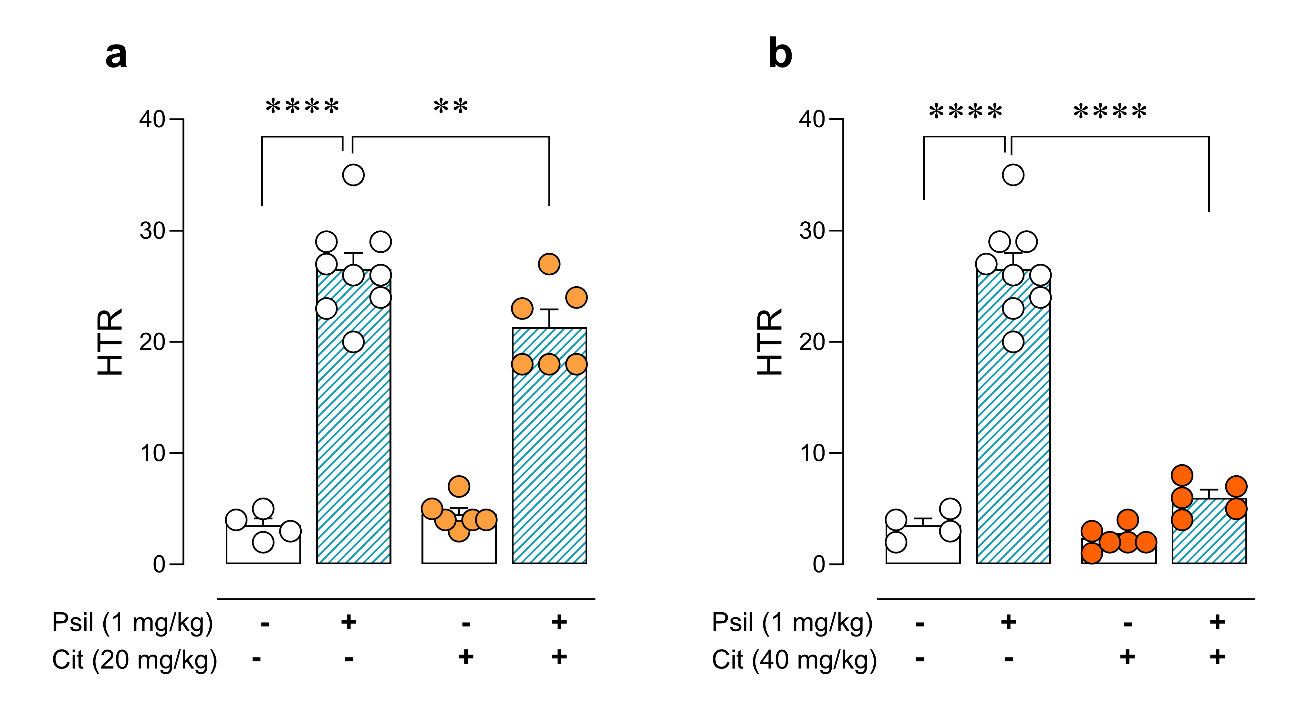
 **a)** Effect of citalopram (20 mg/kg, i.p.) on psilocybin-induced HTR. Two-way ANOVA (F_Psil_(1,21)=203.80, p<0.0001; F_Cit_(1,21)=2.28, p=0.15; F_i_(1,21)=4.96, p<0.05), followed by Bonferroni *post hoc* test (**p<0.01, ****p<0.0001). **b)** Effect of citalopram (40 mg/kg, i.p.) on psilocybin-induced HTR. Two-way ANOVA (F_Psil_(1,20)=117.80, p<0.0001; F_Cit_(1,20)=77.85, p<0.0001; F_i_(1,20)=62.03, p<0.0001), followed by Bonferroni *post hoc* test (****p<0.0001).


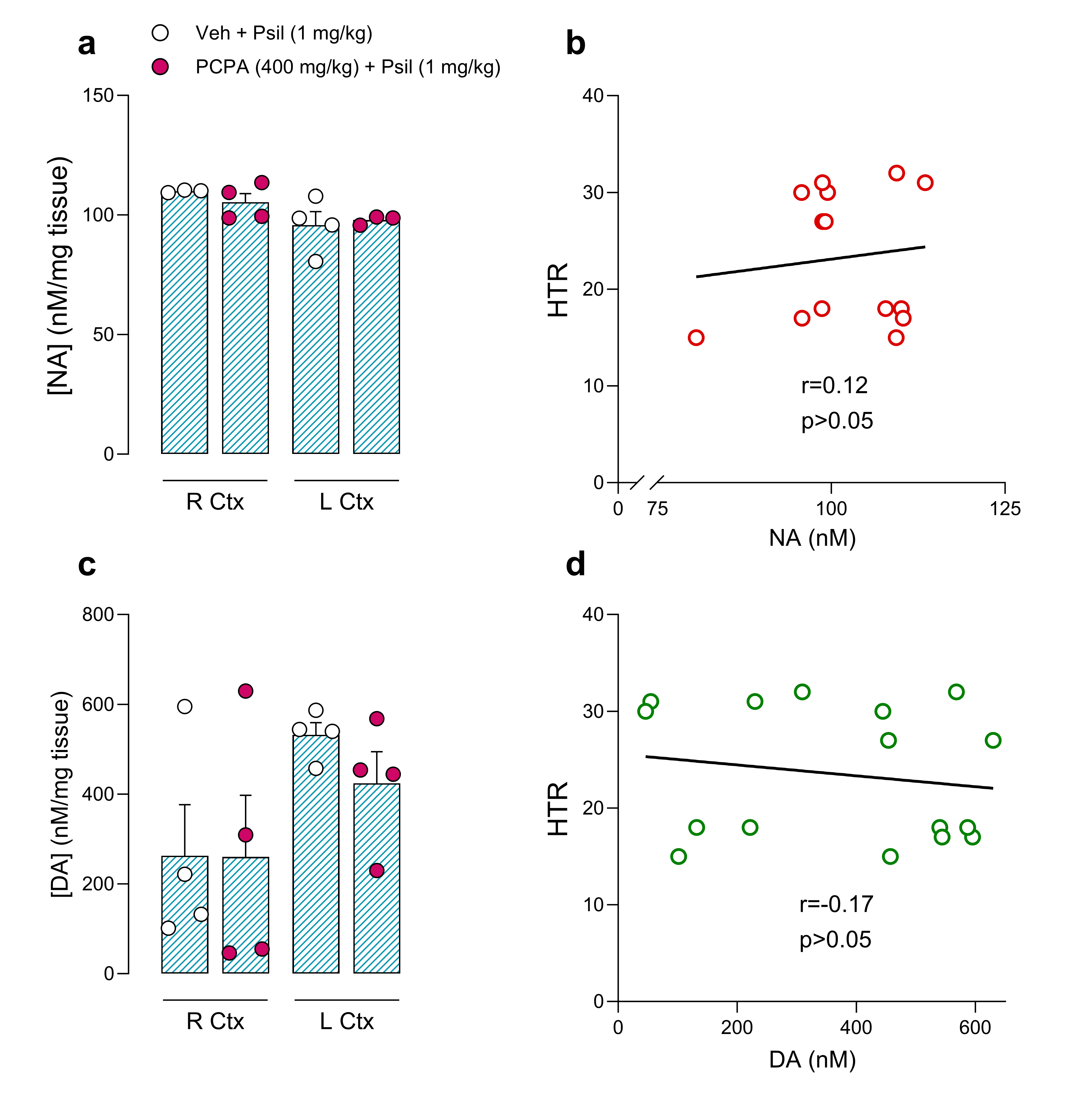


**Figure S3.** Monoamine tissue levels in brain cortex (**left**) and correlation between cortical monoamines and psilocybin (1 mg/kg, i.p.)-induced HTR (**right**) in vehicle / PCPA (400 mg/kg, i.p.) pre-treated mice. **a)** Noradrenaline (NA) concentration in right brain cortex (R Ctx) and left brain cortex (L Ctx) of vehicle/PCPA pre-treated mice **b)** Lack of correlation between cortical NA and psilocybin-induced HTR. **c)** Dopamine (DA) concentration in R Ctx and L Ctx of vehicle/PCPA pre-treated mice. **d)** Lack of correlation between cortical DA and psilocybin-induced HTR.


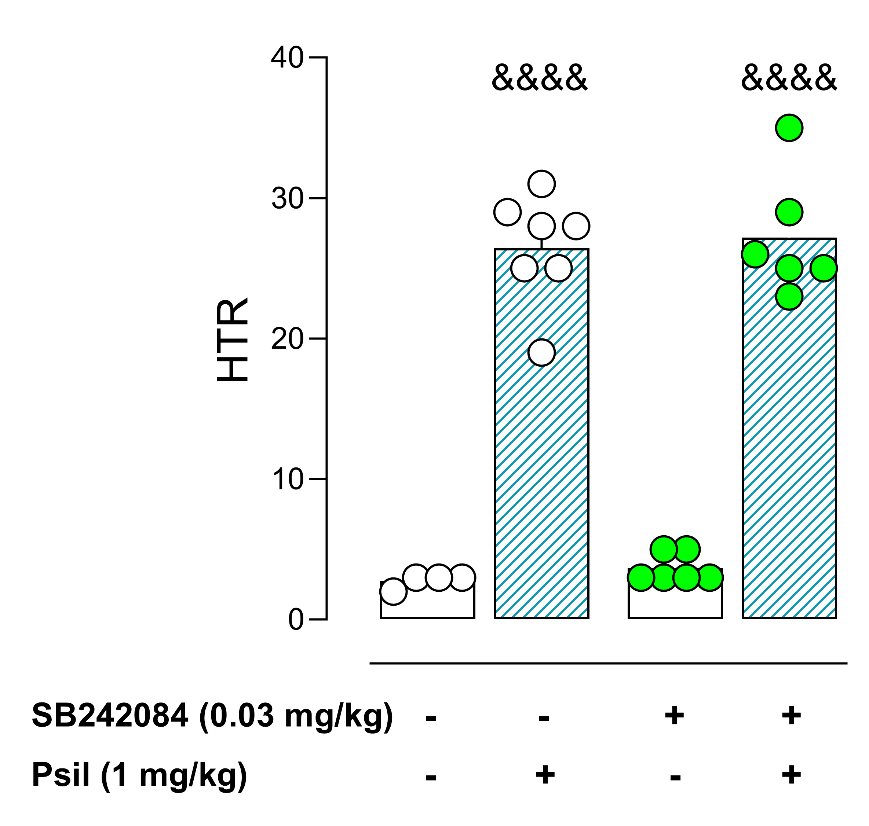


**Figure S4.** Effect of 5HT2CR antagonist SB242084 (0.03 mg/kg, i.p.) on psilocybin (1 mg/kg, i.p.)-induced HTR. Two-way ANOVA. ^&&&&^p<0.0001.
